# Analysis and estimation of cross-flow heat exchanger fouling in phosphoric acid concentration plant using response surface methodology (RSM) and artificial neural network (ANN)

**DOI:** 10.1038/s41598-022-24689-2

**Published:** 2022-11-28

**Authors:** Rania Jradi, Christophe Marvillet, Mohamed Razak Jeday

**Affiliations:** 1 Research Laboratory «Process, Energy, Environment & Electrical Systems», National Engineering School of Gabes, Gabes, Tunisia; 2 CMGPCE Laboratory, French Institute of Refrigeration (IFFI), National Conservatory of Arts and Crafts (CNAM), Paris, France

**Keywords:** Energy grids and networks, Chemical engineering

## Abstract

The production of phosphoric acid by dehydrated process leads to the precipitation of unwanted insoluble salts promoting thus the crystallization fouling build-up on heat transfer surfaces of the exchangers. During the acid concentration operation, the presence of fouling in heat exchangers results in reducing the performance of this equipment, in terms of heat transfer, while increasing energy losses and damaging the apparatus. To mitigate these adverse effects of fouling, it is necessary to forecast the thermal resistance of fouling to schedule and perform exchanger cleaning. In this context, artificial neural network and response surface methodology were used to estimate thermal resistance of fouling in a cross-flow heat exchanger by using the operating data of the concentration loop. The absolute average relative deviations, mean squared errors, root mean squared errors and correlation coefficients were used as indicators error between the experimental and estimated values for both methods. The best fitted model derived from response surface methodology method was second order polynomial while the best architecture topology, for the artificial neural network method, consists of three layers: input layer with six input variables, hidden layer with six hidden neurons and an output layer with single output variable. The interactive influences of operating parameters which have significant effects on the fouling resistance were illustrated in detail. The value of correlation coefficient for the output parameter from the response surface methodology is 0.9976, indicating that the response surface methodology as an assessment methodology in estimating fouling resistance is more feasible compared with the artificial neural network approach.

## Introduction

The deposition of unwanted materials or substances on a heat transfer surfaces which is a more detrimental problem in the industry diminishes the heat transfer and increases the pressure drop of the heat exchangers. This deposit which promotes fouling in the heat exchanger surface, leads to a significant increasing of operation and maintenance costs^1^. Furthermore, due to the expected fouling, the heat exchangers are often oversized for required duty. For these reasons, fouling presents a major challenge in design and operation of heat exchangers for industrialists, designers, technologists and scientists. By decreasing the fouling of heat exchangers, the harmful environmental, economic and humanitarian impacts can be reduced^2^.

The crystallization fouling is one of the detrimental fouling mechanisms in industrial applications. It occurs when dissolved salts precipitate out of the solution due to super saturation. Calcium sulfate is a common salt causing crystallization fouling especially in heat exchangers of the industrial production processes. The inverse solubility of calcium sulfate allows achieving the supersaturated conditions by heating the solution above the limit temperature in which the super saturation occurs or by increasing the concentration by evaporating the solution above the solubility limit^3^. The crystallization fouling of calcium sulfate may cause the increase of the pH, which decreases the solubility of calcium sulfate. The crystallization fouling is strongly attached to the heated surfaces and requires vigorous mechanical or chemical treatment to be removed^2^.

Various parameters can affect crystallization fouling such as thermal conditions of the system, thermodynamics and material properties due to its complexity. Therefore, fouling behavior for the same salt may vary in different systems and operation conditions. In order to reduce the fouling in the process, it is essential to understand the fouling behavior in question^3^.

It was until the 1979s that attention was paid to predict and identify the fouling behavior in order to maintain their undesirable effects at controllable levels. An interesting model for CaSO_4_ fouling of finned tubes during nucleate pool boiling which predict the micro layer super saturation under the bubbles as a function of geometry is presented. The model showed a good agreement with experimental data in the heat flux range of 100–300 kW m^−2^^4^.

The model developed by Jamialahmadi and Müller-Steinhagen which is based on the effects of concentration, surface temperature and fluid velocity, is among the first and rare attempts that mark the history of research of ferocious crystallization fouling and cleaning of calcium phosphate (CaSO_4_) dehydrate, in the view of its negative solubility in phosphoric acid solutions in fertilizer industries^5^. A semi-empirical correlation of crystallization fouling of CaSO_4_ in a rectangular flow channel has been proposed by Mwaba et al. to predict the evolution of the fouling scale layer in heat exchangers in order to assist heat exchanger operators to plan cleaning schedules^6^. The authors have shown that the main parameters which affect the crystallization of calcium sulfate dehydrate on a flat plate were the surface temperature, flow velocity, and degree of super saturation^7^.

Crystallization fouling of calcium sulfate was also investigated in a plate and frame heat exchanger in^8^. The authors studied the effects of flow velocity, wall temperature, and CaSO_4_ concentration on the fouling rates and they observed the distribution of scale along the heat transfer surface.

The studies of Mwaba et al.^6,7^ and of Bansal and Müller-Steinhagen^8^ have shown that crystallization fouling of CaSO_4_ in heat exchanger was surface integration controlled.

In the case of composite fouling; the crystallization can be severely influenced. The most commonly mechanism accompanying crystallization fouling was the particulate fouling^9^. A model for crystallization and particulate fouling prediction at different flow velocities and surface temperatures in plate heat exchangers with and without enhanced heat transfer was developed by Arsenyeva et al.^10^. However, the proposed model was unable to account for salt concentration or solid particle content and sizes. Sheikholeslami^11^ proposed a new model for CaSO_4_ fouling which takes into account the effect of both crystallization and particulate fouling. The developed model was capable to predict the fouling resistance during the cleaning cycle as well as the fouling cycle. From this model, particulate fouling was estimated using the physical mechanism for particle transport and adherence, crystallization was estimated by ionic diffusion, and the removal term was approximated using hydrodynamics of flow and deposit properties. The validation step confirmed a good prediction of the model with literature experimental data. According to the experimental results, it was suggested that crystallization is not the main or only mechanism contributing to CaSO_4_ fouling and particulate fouling seems to be a major contributor.

Estimation of fouling using classical methods has already limitations in terms of accuracy in front of the complexity and the non linearity of the problem^12^. Due to the dramatic advances of information technologies recently, many software as Artificial Neural Network (ANN) could achieve the highly accurate prediction for complex problems^13^. Thereby, this technique can provide useful tools for modeling and correlating practical heat transfer problems. This tool is employed to interpret heat generation/absorption and radiation phenomenon in unsteady electrically conducting Williamson liquid flow along porous stretching surface^14^, to predict the boundary layer flow of a single-walled carbon nanotubes nanofluid toward three different nonlinear thin isothermal needles of paraboloid, cone, and cylinder shapes with convective boundary conditions^15^, to optimize a Darcy–Forchheimer squeezing flow in nonlinear stratified fluid under convective conditions^16^, to model and analyze a mixture of distributions^17,18^ and to predict Soret and Dufour’s convective heat transfer in nanofluid flow through a moving needle^19^.

For fouling phenomenon, an intelligent model is developed for heat exchanger which links fouling resistance to six independent operating parameters of the system (time, fluid density, volume flow rate and inlet and outlet temperatures) by means of Multilayer Perceptron (MLP) network tool^20^. In the same context, for prediction of the fouling factor in both shell-and tube and cross-flow heat exchangers, Jradi et al.^21,22^ used ANN methodology. Other approach has been presented for fouling resistance prediction model for shell and tube heat exchanger using Neural Network method Nonlinear Auto-Regressive with eXogenous as input structure for optimizing operating conditions and preventive maintenance^23^. Furthermore, ANN approach was used to predict the outlet temperature from both shell and tube side of shell and tube heat exchangers in order to planning suitable cleaning schedules^12,24^.

Statistical methods may be an efficient technique to estimate the input–output relationships and to analyze parameter interactions of complex processes^25^. Lately, an increasing pattern of interest has been noticed among the researchers in checking out the suitability of response surface methodology (RSM) and artificial neural network (ANN) modeling approaches to solve problems in a way that fits reality^26^. The RSM and ANN approaches were successfully applied by several researchers in the field of processes modeling^26–29^.

In this context, the present work aimed to analysis and estimates the thermal performance of cross-flow heat exchanger using the RSM and ANN models. Six operating parameters of phosphoric acid concentration loop, comprising acid inlet and outlet temperatures, steam temperature, acid density and acid volume flow and time, are selected as the input parameters, while the fouling resistance is selected as the output parameter. In addition, the functions between the input and output parameters are obtained using both the RSM and ANN models. A comparison of these two models is also presented based on the estimation functions of the heat exchanger. These models provide effective approaches for estimating and optimizing the thermal performances accurately and quickly.

## Methodology

The objective of this article is to apply two different methods to estimate the fouling which are the RSM and ANN and to compare between these two techniques. This analysis consists of three main parts: (1) Process description of the industrial phosphoric acid concentration unit. (2) Experimental procedure to calculate the fouling resistance. (3) Mathematical modeling of the fouling resistance.

### Experimental process description

The process used for the production of phosphoric acid is a dehydrate process which comprises a stage of phosphoric acid concentration. This last stage consists in passing P_2_O_5_ from a range of 28–32% to a range of 40–54%, this is done by evaporation of the water.

**Figure 1:**
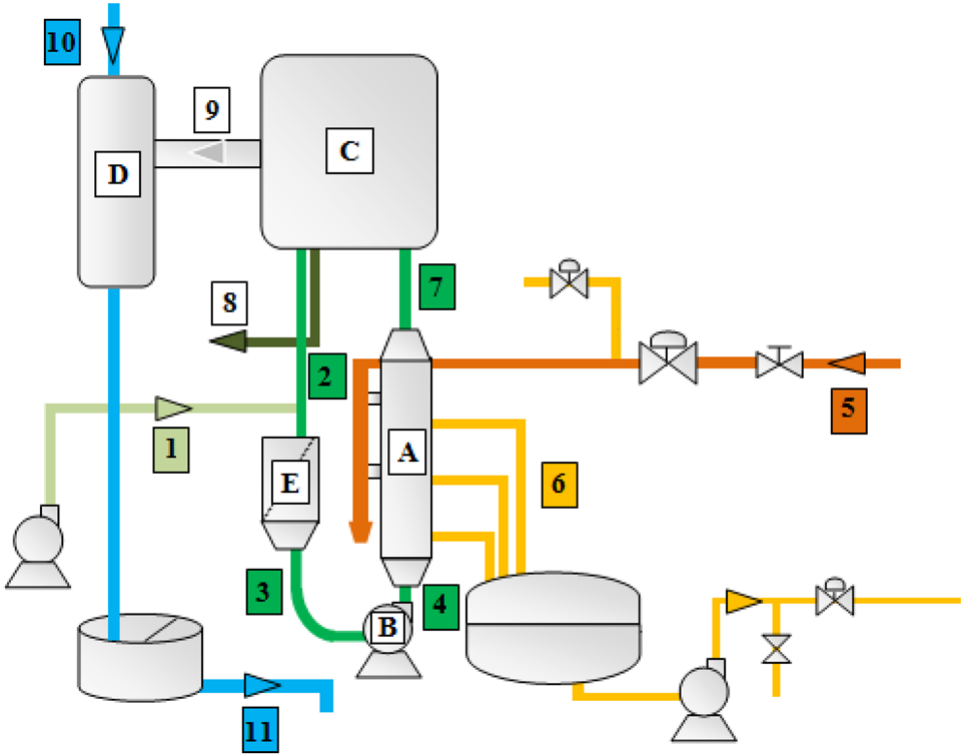
Schematic drawing of the phosphoric acid concentration process.

Figure 1 presents the flow diagram of phosphoric acid concentration unit. The concentration of phosphoric acid is produced by “Rhone Poulenc” process flows in a closed loop forced-circulation evaporator, operating under vacuum ensured by a barometric condenser. As can be seen from Fig. 1, this evaporator consists of different equipment: a cross-flow heat exchanger (**A**), a centrifugal pump (***B***), a boiler or expansion chamber (***C***), a barometric condenser (***D***) and a basket filter (***E***)^20^.

The addition of dilute phosphoric acid (28% P_2_O_5_) (1) takes place at the basket filter where it mixes with the circulating phosphoric acid (2) in order to retain crusts and gypsum debris which may be hang up on the circulation pump and risk blocking the heat exchanger tubes. Therefore, the basket filter protects the pump from abrasion and limits clogging in the heat exchanger tubes. This makes it possible to minimize the frequency of stopping for washing.

The circulation pump aspirates the blend (3) formed, freed of coarse impurities, and send it to the heat exchanger.

In the cross-flow heat exchanger, the circulating phosphoric acid (4) is introduced into the graphite tubes at a temperature of about 70 °C to raise it to about 80 °C and the steam (5), which undergoes a condensation at a temperature of 120 °C, circulates perpendicularly around them. Steam distribution and condensate recovery are done through the use of a steel shell with baffles. These baffles ensure the positioning of the blocks and force the steam to pass through the holes and ensure the recovery of condensate. The condensate (6) is sent to a storage tank before being sent back to the utility station. The superheated mixture of phosphoric acid (7) leaves the exchanger then passes into the boiler where a quantity of water evaporates at the boiling point and the concentrated acid (8) is produced by overflowing in a piping system inside the boiler. The remaining amount of phosphoric acid is recycled. The condenser also ensures incurring non-condensable gases coming out from the boiler (9) by the effect of a hydro-ejector valuing the relaxation of pressurized water flow (10). At the foot of the barometric guard, the sea water is recovered in a tank of guard before being released into the sea.

The cleaning operation of the phosphoric acid concentration loop is carried out using sea water for 8 h to guarantee that the heat exchanger is totally free of fouling at the beginning of a new run. Each operating cycle lasts an average of 5 days and interruptions are sometimes necessary for mechanical interventions.

The choice of heat exchanger construction materials depends on the corrosiveness and scaling characteristics of phosphoric acid. The graphite blocks heat exchanger offers a very high thermal conductivity and it is totally resistant to corrosion, but it is a relatively fragile material and thus can lead to operating problems due to tube breakage during frequent cleaning or bad-operation^30^.

### Experimental procedure

Totally, seven heat exchangers distributed between three units for the concentration of phosphoric acid are contained at the phosphoric acid production plant of the Tunisian Chemical Group. Three of them are of type graphite blocks. The specific choice of this heat exchanger, whose main characteristics are listed in Table 1, is based on the large production capacity and the possibility to collect a sample of fouling for identification during data collection.

**Table 1:** Main characteristics of the studied heat exchanger.

Main characteristics	Graphite blocks
Number of blocks	11
Blocs height (m)	0.513
Acid-side tube length (m)	5.564
Steam-side tube length (m)	1.025
Diameter of acid-side duct (mm)	16
Diameter of steam-side duct (mm)	15
Exchange surface (m^2^)	249.5
Number of acid-side ducts	868
Number of steam-side ducts	483

The experimental calculation of the overall heat transfer coefficient (U_t_) and the fouling resistance (R_f_) is based on the following simplifying assumptions:

- The flows of two fluids (phosphoric acid and steam) are at counter current.
- Thermal losses are neglected.
- Condensation of the superheat steam is total.
- Fouling is only formed in phosphoric acid side.

The inlet and outlet temperatures of the two fluids, the suction and discharge pressure of the pump are taken respectively at the extremities of the heat exchanger and of the circulation pump while the density of the flow phosphoric acid is measured at the inlet of the heat exchanger. The frequency of data acquisition was of two hours. The setup was equipped with a data acquisition system composed by a computer and a data acquisition card (high-speed analog-to-digital converter card). The temperatures of the phosphoric acid and the steam at the inlet and outlet of the heat exchanger were measured using three thermocouples type Pt100 class with the uncertainty of ± 0.3 °C.

Moreover, the density of the phosphoric acid was measured using a Densimeter DMA35 with the uncertainty of ± 0.05%. Besides, a Diaphragm pressure gauge with the uncertainty of ± 1.6% was used to measure the pressure.

The flow rate of phosphoric acid, passing through the exchanger can be calculated from the suction and discharge pressures and pump’s characteristic curve^31^.

The parametric range of operating variables corresponding to the heat exchanger used in this unit is listed in Table 2.

**Table 2:** Parametric ranges.

Variable	Unit	Designation	Measurement ranges
Acid inlet temperature	°C	T_in,ac_	68–78
Acid outlet temperature	°C	T_out,ac_	77–86.8
Steam temperature	°C	T_st_	116–125
Suction pressure	bar	P_suc_	0.85–1.25
Discharge pressure	bar	P_dis_	3.1–3.9
Acid density	Kg/m^3^	ρ_ac_	1620–1656

Using the energy balance, the heat flow ($Q_{ac}$) transmitted from steam to phosphoric acid is given by following Eq. (1)^31^:

1 $$Q_{ac} = \dot{m}_{ac,cir} \times Cp_{ac} \times (T_{out,ac} - T_{in,ac} )$$

where $\dot{m}_{ac,cir}$, T_in, ac_, T_out,ac_ and Cp_ac_ are the mass flow rate, inlet and outlet temperatures and the specific heat capacity of phosphoric acid, respectively.

The overall heat transfer coefficient under fouling condition (U_t_) is defined as^31^:

2 $$U_{t} = \left( {\frac{Q}{{A \times \Delta T_{lm} \times F}}} \right)_{t}$$

The Logarithmic Mean Temperature Difference (ΔT_lm_) is defined as^31^:

3 $$\Delta T_{lm} = \frac{{T_{in,ac} - T_{out,ac} }}{{\ln \left\{ {\frac{{(T_{st} - T_{in,ac} )}}{{(T_{st} - T_{out,ac} )}}} \right\}}}$$

where A is the heat transfer area, F is the corrective factor for the average logarithmic temperature difference (= 1 pure Counter Flow Arrangement), T_in, ac_ and T_out, ac_ are inlet and outlet acid temperatures and T_st_ is the steam inlet temperature.

The studied heat exchanger underwent mechanical cleaning operation between operating runs. In this case, it is totally free of fouling at the beginning of each new run. The overall heat transfer coefficient at the beginning of every cycle is considered as the value of the clean design (U_t=0_).

The fouling resistance according to time (R_f_) is then given by Eq. (4)^1,20^:

4 $$R_{f} = \frac{1}{{U_{t} }} - \frac{1}{{U_{t = 0} }}$$

### Mathematical models

#### Response surface methodology

Response surface methodology (RSM) is a tool of mathematical, theoretical and statistical techniques used to finding the relationship between several independent variables and one or more responses^32^. Since 1951when Box and Wilson developed this method, it has been used widely as a technique for designing experiments. The base of this method is to fitting the mathematical models to the experimental results generated from the designed experiment and to verifying the model obtained by means of statistical techniques^29^.

It is a fundamental tool in the field of engineering used for developing, improving, and optimizing issues where a response variable is influenced by multiple influencing variables^26^. Design Expert Statistical Software package 10 is used in this studied to develop the RSM model. The Central Composite Design (CCD) in RSM which provides high quality predictions is a fractional factorial design method used to find the functional relationship between the expected response and the input variables as shown in Eq. (5)^26^.

5 $$Y = f(X_{1} ,X_{2} ....,X_{n} )$$

where Y represents the response of the system, f represents the unknown of the response, X_1_, X_2_,…, X_n_ represent the actual independent process variables and n is the number of independent variables.

A face-centered central composite design was constructed with a value of (α) equal to 1 for six factors and each factor has three levels (Table 3).

**Table 3:** Factors and factor levels for RSM.

Factor	Code	Factors level of code		
		Low level − 1	Intermediate level 0	High level + 1
T (h)	A	0	61	122
T_in,ac_ (°C)	B	68	73	78
T_out,ac_ (°C)	C	77	81.9	86.8
T_st_ (°C)	D	116	120.5	125
ρ_ac_ (Kg/m^3^)	E	1620	1638	1656
$\dot{v}_{ac,cir}$(m^3^/h)	F	2102	2754.5	3407

The number of experiments needed can be calculated by following Eq. (6)^26^.

6 $$N = 2^{z} + 2z + n$$

where z is the number of factors, and n is the number of center points.

The general order polynomial regression equation that defines the relationship between the model response (Y) and the process parameters (X_i_) is given by Eq. (7)^26^.

7 $$Y = \gamma_{0} + \sum\limits_{i = 1}^{n} {\gamma_{i} } X_{i} + \sum\limits_{i = 1}^{n} {\gamma_{ii} } X_{i}^{2} + \sum\limits_{i = 1}^{n} {\sum\limits_{j = 2}^{n} {\gamma_{ij} } X_{i} } X_{j} ;(i \ne j)$$

where γ_0_ is the constant coefficient, γ_i_, γ_ii_, and γ_ij_ are the interaction coefficients of the linear, quadratic and second order term, respectively. The X_i_ and X_j_ are the process parameters.

The regression equation of the response, analysis of variance (ANOVA) and interactive effects analysis of the different variables are the three main analytical steps of RSM analysis.

#### Artificial neural network

The use of Artificial Neural Networks (ANN) started a century ago, conceptually and structurally inspired from the capabilities exhibited by biological neural systems^33,34^. This learning capacity with training data makes ANN more powerful than the parametric approaches because of its ability to model a multivariable problem containing complex relationships between the variables and to extract implied non-linear relationships between these variables. This technology is becoming increasingly applicable as a promising tool. It offers an alternative way for solving nonlinear and complex problems in actual situations in various engineering fields, for instance, the control of dynamic and aging processes in heat transfer such as fouling^35^.

ANN models are generally constituted of three main layers, namely the input, hidden, and output layers. Each layer has a specific numbers of neurons. The size of the input and output layers are always equal respectively to the number of independent and dependent variables.

Simultaneous choice of the optimal number of hidden layers and the number of neurons in hidden layer is a very important step to set overall neural network architecture. The whole of hidden layers interact indirectly with the external source but have enormous influence on the final output^36^. Various approaches were proposed by several researchers to determine the number of hidden layer in neural network.

Choosing one hidden layer for estimating the fouling resistance for the studied heat exchanger comes from the work of Cybenko and Hornik in 1989 who proved that the use of three layers network (with one input layer, one output layer, and one hidden layer) can simulate any complex nonlinear problems^37,38^.

The selection of hidden layer size is a specific problem and there is no general rule for determining this number. The number of neurons in hidden layer, to some extent, depends on the number and the quality of training patterns. This number must be sufficient for correct modeling of the problem as well as it should be low to ensure generalization.

Several studies were done to determine the number of neurons in the hidden layer^39,40^. In this work, to establish a suitable and stable network for the problem; many networks are built by changing their size in order to reach a suitable result.

The summation and activation functions are the two types of functions which can be used for each layer. The choice of the use of activation function in this studied is based on the ability of this function to determine the output of the cell which provides a suitable match between the input and output layers. By contrast, the summation function is used to obtain only the net input of a cell. The appropriate choice of the activation function can have a profound influence on network performance. For this reason, it is important to select the better activation function to have an easier and faster convergence of neural networks^41^. Therefore, there are different types of transfer functions, the most frequent used are the linear or identity transfer function, the logistic or sigmoid transfer function and the hyperbolic tangent transfer function. Several studies in this field of heat exchanger fouling confirm that the hyperbolic transfer function is the best performed transfer function^20^. In this study, three transfer functions mentioned previously are adopted for the hidden and output layers of the ANN model and a comparison among them is carried out.

Among different applications, the back-propagation (BP) training algorithm including BFGS (Broyden–Fletcher–Goldfarb–Shanno) and Scaled Conjugate Gradient methods are usually adopted to find the optimum parameter values of the neural networks. The performance of these methods is significantly better than traditional techniques such as Gradient Descent. But they have more intensive memory and computational demanding generally. However, these techniques may require a small number of iterations to train a neural network by considering their fast convergence rate^42,43^. Therefore, the BP neural network with BFGS, Scaled Conjugate Gradient and Gradient Descent training functions are thus used in this work and a comparison among them is carried out.

#### Performance of RSM and ANN models

The performance of the RSM and ANN models was evaluated by using four statistical parameters which are: the absolute average relative deviation (AARD %), the mean squared error (MSE), the root mean squared error (RMSE) and the correlation coefficient (r^2^). The mathematical equations Eqs. (8)–(11) of these parameters are given below^20,44,45^:

8 $$AARD\% = \frac{100}{N}\sum\limits_{i = 1}^{N} {\frac{{\left| {R_{fi}^{\exp } - R_{fi}^{pred} } \right|}}{{R_{fi}^{\exp } }}}$$

9 $$MSE = \frac{1}{N}\sum\limits_{i = 1}^{N} {(R_{fi}^{\exp } - R_{fi}^{pred} )^{2} }$$

10 $$RMSE = \sqrt {\frac{{\sum\limits_{i = 1}^{N} {(R_{fi}^{\exp } - R_{fi}^{pred} )^{2} } }}{N}}$$

11 $$r^{2} = \frac{{\sum\limits_{i = 1}^{N} {(R_{fi}^{\exp } - \overline{{R_{f} }} )^{2} - \sum\limits_{i = 1}^{N} {(R_{fi}^{\exp } - R_{fi}^{pred} )^{2} } } }}{{\sum\limits_{i = 1}^{N} {(R_{fi}^{\exp } - \overline{{R_{f} }} )^{2} } }}$$

where R_f_ represents the fouling resistance. R_f_^exp^ and R_f_^pred^ represent the experimental and predicted fouling resistance. $\overline{{R_{f} }}$ is the average value of the experimental fouling resistance. N is the number of value pairs used for the comparison of methods.

## Results and discussion

### RSM analysis

#### Regression equation of response

By using the Design Expert Statistical Software package 10 and according to the CCD, this investigation was executed to study the influence of the parameters [time (t), acid inlet (T_in, ac_) and outlet (T_out, ac_) temperatures, steam temperature (T_st_), acid density (ρ_ac_) and acid volume flow ($\dot{v}_{ac,cir}$)] and to predict the fouling resistance (R_f_). The quadratic equation was developed by utilizing experimental results to express the response with respect to (t, T_in, ac_, T_out, ac_,T_st_, ρ_ac_ and $\dot{v}_{ac,cir}$).

In terms of coded factors, the model equation Eq. (12) is depicted below:

12 $$\begin{aligned} Y &= 8.363 \times 10^{ - 5} + 1.016 \times 10^{ - 5} A + 1.860 \times 10^{ - 4} B - 2.266 \times 10^{ - 4} C \\ & \quad + \;3.732 \times 10^{ - 5} D - 1.982 \times 10^{ - 6} E - 8.851 \times 10^{ - 5} F + 2.011 \times 10^{ - 6} AB \\ &\quad - \;3.416 \times 10^{ - 6} AC + 3.916 \times 10^{ - 6} AD + 5.641 \times 10^{ - 6} AE - 2.145 \times 10^{ - 6} AF \\ &\quad - \;2.765 \times 10^{ - 4} BC + 2.565 \times 10^{ - 5} BD - 5.382 \times 10^{ - 5} BE - 7.670 \times 10^{ - 5} BF \\ &\quad - \;1.780 \times 10^{ - 5} CD + 5.322 \times 10^{ - 5} CE + 9.158 \times 10^{ - 5} CF - 9.853 \times 10^{ - 7} DE \\ &\quad - \;9.052 \times 10^{ - 7} DF + 1.867 \times 10^{ - 5} EF - 3.915 \times 10^{ - 6} A^{2} + 1.203 \times 10^{ - 4} B^{2} \\ &\quad + \;1.470 \times 10^{ - 4} C^{2} + 5.573 \times 10^{ - 7} D^{2} + 1.607 \times 10^{ - 7} E^{2} + 2.756 \times 10^{ - 5} F^{2} \\ \end{aligned}$$

**Figure 2:**
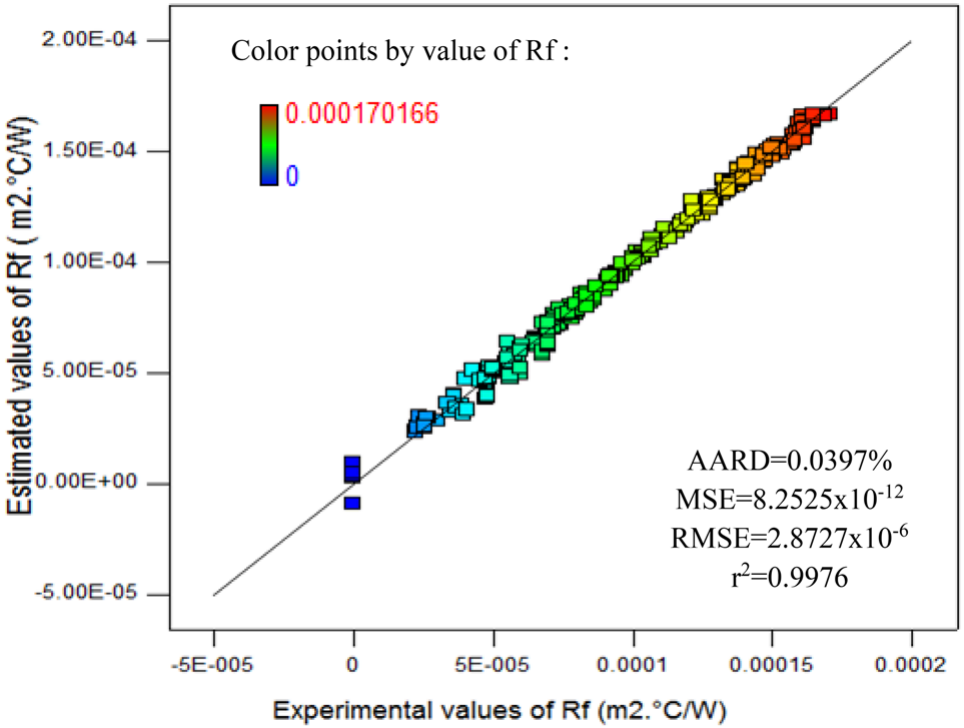
RSM estimation versus experimental results.

The deviation between the experimental results and values estimated by the RSM for fouling resistance (R_f_) is indicated in Fig. 2. The color points in Fig. 2 refer to the high and low values of fouling resistance. It can be observed from the graph that the concentration of the experimental points is approximately along the 45° line, which reveals the significance of the regression model. This observation indicates that the CCD is well fitted into the model, thus it can be applied to perform the optimization operation of the process.

The r^2^ for (R_f_) is 0.9976, while the values of the absolute average relative deviation, mean squared error and root mean squared error for the response are very low.

As mentioned in Table 2, the operating periods ranging should be up to 122 h, the inlet and outlet temperatures of fluid and the steam temperature should not exceed 78 °C, 86.8 °C and 125 °C, respectively, the phosphoric acid density should not be above 1656 kg/m^3^, and the volume flow rate should not be below 2102 m^3^/h to obtain values of (R_f_) close to reality by the developed model.

#### Analysis of variance (ANOVA)

The “analysis of variance test” widely known as ANOVA is the most efficient method that gives an idea about statistical significance of the quadratic regression model equation, the independent parameters, their interactions, and the goodness of fit^46^.

The model fitness quality was assessed at the 5% significance level by p-value (probability value), F-value (Fisher’s test), R^2^ and R^2^_adjusted_^47^. The results of ANOVA are displayed in Table 4.

**Table 4:** Analysis of variance of the RSM model for output parameter.

Source	Sum of squares	Degrees of freedom	F value	p-value
Model	6.454 × 10^–07^	27	2671.84	< 0.0001
A	6.508 × 10^–11^	1	7.27	0.0074
B	2.728 × 10^–09^	1	304.89	< 0.0001
C	2.875 × 10^–09^	1	321.37	< 0.0001
D	1.533 × 10^–09^	1	171.36	< 0.0001
E	7.178 × 10^–12^	1	0.80	0.3710
F	3.088 × 10^–09^	1	345.12	< 0.0001
AB	5.503 × 10^–14^	1	6.151 × 10^–03^	0.9375
AC	1.127 × 10^–13^	1	0.013	0.9107
AD	3.525 × 10^–12^	1	0.39	0.5306
AE	1.143 × 10^–11^	1	1.28	0.2593
AF	3.459 × 10^–13^	1	0.039	0.8442
BC	7.138 × 10^–11^	1	7.98	0.0050
BD	1.839 × 10^–11^	1	2.06	0.1526
BE	1.048 × 10^–10^	1	11.72	0.0007
BF	3.756 × 10^–11^	1	4.20	0.0412
CD	6.258 × 10^–12^	1	0.70	0.4036
CE	7.222 × 10^–11^	1	8.07	0.0048
CF	3.781 × 10^–11^	1	4.23	0.0406
DE	6.559 × 10^–13^	1	0.073	0.7867
DF	1.309 × 10^–13^	1	0.015	0.9038
EF	5.476 × 10^–11^	1	6.12	0.0139
A^2^	7.926 × 10^–12^	1	0.89	0.3473
B^2^	7.562 × 10^–11^	1	8.45	0.0039
C^2^	5.660 × 10^–11^	1	6.33	0.0124
D^2^	4.206 × 10^–13^	1	0.047	0.8285
E^2^	5.684 × 10^–14^	1	6.353 × 10^–03^	0.9365
F^2^	8.457 × 10^–11^	1	9.45	0.0023
Residual	2.979 × 10^–09^	333		
Corrected Total	6.484 × 10^–07^	360		
R^2^	0.9954			
R^2^_predicted_	0.9932			
R^2^_adjusted_	0.9950			
Adequate Precision	211.574			

In statistics, the p-value is employed to verify the significance of the model or model term. If the p value is less than 0.05, this implies that the factors show significance for the response^47^. If the F-value is higher than 0.05 for the independent process variable, the effect of that variable become higher^47^. In these cases, the model can be applied to estimate results accurately.

From the ANOVA results, the derived model is statistically significant at 95% confidence level since the p-value is much lower than 0.05 (p-value = 0.0001) and the F-value is equal to 2671.84.

Based on the calculated p-values (< 0.0001) as shown in Table 5, the main effects of the time (A), acid inlet and outlet temperatures (B and C), steam temperature (D) and acid volume flow (F), as well as their interactions BC, BE, BF, CE, CF and EF, and quadratic terms B^2^, C^2^, and F^2^ are statistically significant for the fouling resistance (R_f_), but the linear term (acid density (E)) is not significant with P and F-values of 0.3710 and 0.80. The other mix product and quadratic terms with larger p value have minor effects on (R_f_).

**Table 5:** Input and output parameters used by ANN method.

Parameters	Variable	Unit	Designation	Measurement ranges
Input	Time	h	t	0–122
	Acid inlet temperature	°C	T_in,ac_	68–78
	Acid outlet temperature	°C	T_out,ac_	77–86.8
	Steam temperature	°C	T_st_	116–125
	Acid density	Kg/m^3^	ρ_ac_	1620–1656
	Acid volume flow rate	m^3^/h	$\dot{v}_{ac,cir}$	2102–3407
Output	Fouling resistance	m^2^.°C/W	R_f_	0–0.00017

The determination coefficient (R^2^) is a measure of how efficient the variability in the measured output can be explained by the experimental variables and their interactions; therefore, it is regarded as the degree of model fitness^47^. In this case, the R^2^ is 0.9954 which indicates that the adjustment of the quadratic model to the experimental results is satisfactory since it is near to unity.

The R^2^_predicted_ and R^2^_adjusted_ gives an idea about the quality or adequacy of the model^47^. The difference between the R^2^_predicted_ and R^2^_adjusted_ should be less than 0.02 to consider that the model is adequate^47^.

In this study, the model which describes the experimental design response is adequate, since the difference between R^2^_predicted_ and R^2^_adjusted_ is 0.0018. From Table 4, the value of R^2^_adjusted_ is equal to 0.9950 which indicates that the model fits the experimental data satisfactorily.

The adequate precision is a measure of the signal-to-noise ratio^47^. A greater value of this statistical parameter than 4 is desirable for a good model. As shown in Table 4, the adequate precision is 211.574 which indicates that the signal is adequate and the quadratic model can be used to navigate the designed space^47^.

**Figure 3:**
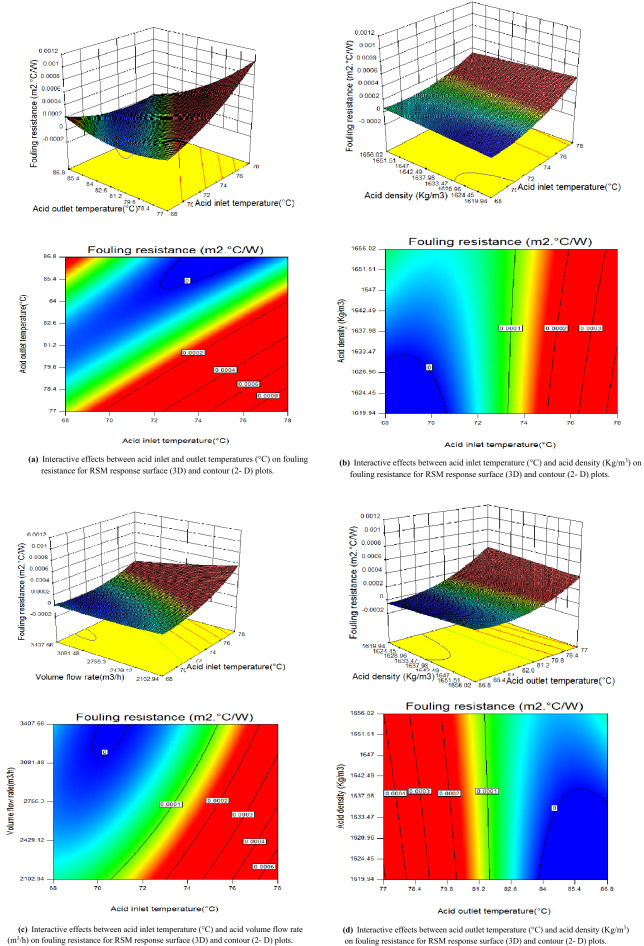

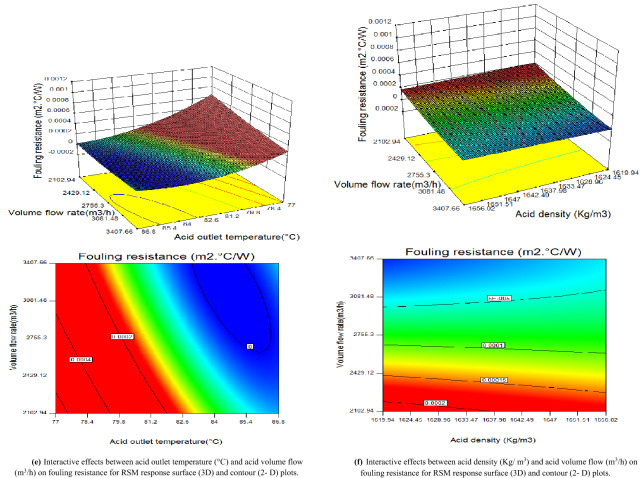
(**a**) Interactive effects between acid inlet and outlet temperatures (°C) on fouling resistance for RSM response surface (3D) and contour (2-D) plots. (**b**) Interactive effects between acid inlet temperature (°C) and acid density (Kg/m^3^) on fouling resistance for RSM response surface (3D) and contour (2-D) plots. (**c**) Interactive effects between acid inlet temperature (°C) and acid volume flow rate (m^3^/h) on fouling resistance for RSM response surface (3D) and contour (2-D) plots. (**d**) Interactive effects between acid outlet temperature (°C) and acid density (Kg/m^3^) on fouling resistance for RSM response surface (3D) and contour (2-D) plots. (**e**) Interactive effects between acid outlet temperature (°C) and acid volume flow (m^3^/h) on fouling resistance for RSM response surface (3D) and contour (2-D) plots. (**f**) Interactive effects between acid density (Kg/ m^3^) and acid volume flow (m^3^/h) on fouling resistance for RSM response surface (3D) and contour (2-D) plots.

The RSM is an adequate method due to its flexibility in design space navigation. The 2-D and 3-D surface response plots explore the designed space and predict the optimal conditions of the fouling resistance. According to the p values in Table 4, the interactions of BC, BE, BF, CE, CF and EF have significant influences on the fouling resistance; thus, these six sets of interactions are studied in detail, as shown in Fig. 3a–f.

#### Interactive effects analysis

By using RSM, six sets of 2-D and 3-D surface plots are drawn to study the interactions of the four independent parameters which are acid inlet (T_in, ac_) and outlet (T_out, ac_) temperatures, acid density (ρ_ac_) and acid volume flow ($\dot{v}_{ac,cir}$).

These 3-D and 2-D contour plots show the interactive effects between: acid inlet and outlet temperatures (Fig. 3a), acid inlet temperature and acid density (Fig. 3b), acid inlet temperature and acid volume flow (Fig. 3c), acid outlet temperature and acid density (Fig. 3d), acid outlet temperature and acid volume flow (Fig. 3e) and acid density and volume flow (Fig. 3f), respectively on the fouling resistance.

From the global 3-D graphs, some degrees of curvature are seen which can be attributed to the reflection of the levels of uncertainties associated with every interaction of the process variables.

It can be seen from Fig. 3a that the fouling resistance increases linearly with the acid inlet temperature. As the acid outlet temperature decreases, the fouling resistance decreases up to a specific level then increases. Moreover, the maximal values of the fouling resistance can be obtained by decreasing their acid outlet temperature and increasing the acid inlet temperature.

The interactive influences of the acid inlet temperature and acid density on the fouling resistance are presented in Fig. 3b. It can be seen that the fouling resistance increases proportionally with the acid inlet temperature. Furthermore, the acid inlet temperature has a larger effect than the acid density. This is because the fouling resistance is determined by the temperatures of the two fluids in the studied heat exchanger based on the logarithmic mean temperature difference. Although the acid density affects the fouling resistance, it is not the main factor.

As seen in Fig. 3c, the fouling resistance increases with the acid inlet temperature which is confirmed with the previous results. In addition, the fouling resistance increases by decreasing the volume flow rate. When the acid volume flow rate is fixed to 2103 m^3^/h; and for an acid inlet temperature of 78 °C, the maximal fouling resistance is 0.00058 m^2^ °C/W.

As seen from Fig. 3d, as the acid outlet temperature decreases, the fouling resistance increase and display nonlinear variations. Additionally, the acid outlet temperature has a significant effect on the fouling resistance, while the acid density has a minor effect which is confirmed with Fig. 3b.

For a small acid volume flow rate, the fouling resistance increases by decreasing the acid outlet temperature (shown in Fig. 3e). The acid volume flow rate is not the main factor that affects the fouling resistance.

As seen in Fig. 3f, a small variation of acid volume flow and acid density can increase fouling resistance. The fouling resistance reaches 0.0002 m^2^ °C/W when the acid volume flow and acid density are equal to 2103 m^3^/h and 1620 kg/m^3^, respectively. This is because the fouling resistance is inversely proportional to the acid volume flow rate, and can be increased by decreasing the volume flow. However, the decrease in the volume flow rate results in the perturbation and instability due to the fouling phenomena in the heat exchanger; thus, the appropriate volume flow should be selected for a reasonable range of design.

### ANN analysis

#### ANN architecture

In this section, ANN model was developed utilizing feed-forward back propagation neural network as mentioned previously to estimate the fouling resistance in a cross-flow heat exchanger. In modeling and according to the default percentages of divider and function in STATISTICA software, 70% of the data set was employed for the training set, whereas the remaining 30% of the data set was for the validation and testing set (15% each). The data set correspond to 7 operating cycles containing a total of 361 observations. Time (t), acid inlet (T_in, ac_) and outlet (T_out, ac_) temperatures, steam temperature (T_st_), acid density (ρ_ac_) and acid volume flow ($\dot{v}_{ac,cir}$) were considered as input parameters and fouling resistance was as output parameter. The measurement ranges of input and output parameters are depicted in Table 5.

In a single hidden layer, the number of neurons was varied from one to twelve to determine the adequate ANN configuration for fouling resistance prediction.

Due to the randomness of NN training caused by data set splitting or learning iteration, cross validation should be required^48^. For this reason, thirty various trained network, was tested and validated and the best performance among trained network for each topology is listed in Table 6. So, theoretically the optimal ANN configuration selected is based on the least prediction of validation errors^24,49^.

**Table 6:** Comparison of errors of several ANN configurations for estimation of fouling resistance.

ANN configuration	r^2^_ALL_	Training error				Validation error				Test error			
		AARD	MSE	RMSE	r^2^	AARD	MSE	RMSE	r^2^	AARD	MSE	RMSE	r^2^
6–1–1	0.9848	0.0864	5.5236 × 10^–11^	7.4321 × 10^–06^	0.9849	0.1040	6.8054 × 10^–11^	8.2495 × 10^–06^	0.9847	0.1069	6.2149 × 10^–11^	7.8834 × 10^–06^	0.9873
6–2–1	0.9872	0.0812	4.4658 × 10^–11^	6.6826 × 10^–06^	0.9876	0.0968	5.9795 × 10^–11^	7.7327 × 10^–06^	0.9862	0.1005	5.0716 × 10^–11^	7.1215 × 10^–06^	0.9894
6–3–1	0.9914	0.0514	3.1604 × 10^–11^	5.6217 × 10^–06^	0.9914	0.0651	3.9511 × 10^–11^	6.2858 × 10^–06^	0.9910	0.0396	2.8074 × 10^–11^	5.2985 × 10^–06^	0.9927
6–4–1	0.9916	0.0594	2.8458 × 10^–11^	5.3346 × 10^–06^	0.9919	0.0835	3.5984 × 10^–11^	5.9987 × 10^–06^	0.9913	0.0750	3.3369 × 10^–11^	5.7766 × 10^–06^	0.9922
6–5–1	0.9931	0.0453	2.1807 × 10^–11^	4.6698 × 10^–06^	0.9937	0.0875	3.4220 × 10^–11^	5.8498 × 10^–06^	0.9919	0.0772	2.9697 × 10^–11^	5.4495 × 10^–06^	0.9927
**6–6–1**	**0.9950**	**0.0390**	**1.6685 × 10**^**–11**^	**4.0848 × 10**^**–06**^	**0.9952**	**0.0793**	**2.5853 × 10**^**–11**^	**5.0846 × 10**^**–06**^	**0.9938**	**0.0591**	**1.7071 × 10**^**–11**^	**4.1318 × 10**^**–06**^	**0.9958**
6–7–1	0.9855	0.0839	5.2792 × 10^–11^	7.2658 × 10^–06^	0.9853	0.0940	6.2538 × 10^–11^	7.9081 × 10^–06^	0.9851	0.0897	5.0535 × 10^–11^	7.1088 × 10^–06^	0.9889
6–8–1	0.9931	0.0441	2.2292 × 10^–11^	4.7214 × 10^–06^	0.9936	0.0827	3.3818 × 10^–11^	5.8154 × 10^–06^	0.9919	0.0733	2.8552 × 10^–11^	5.3434 × 10^–06^	0.9929
6–9–1	0.9886	0.0648	4.0142 × 10^–11^	6.3358 × 10^–06^	0.9887	0.0909	5.2919 × 10^–11^	7.2745 × 10^–06^	0.9874	0.0801	4.0283 × 10^–11^	6.3469 × 10^–06^	0.9916
6–10–1	0.9889	0.0630	3.9534 × 10^–11^	6.2876 × 10^–06^	0.9893	0.0893	5.3437 × 10^–11^	7.3101 × 10^–06^	0.9876	0.0777	4.6184 × 10^–11^	6.7959 × 10^–06^	0.9906
6–11–1	0.9899	0.0477	3.4877 × 10^–11^	5.9056 × 10^–06^	0.9900	0.0920	4.5248 × 10^–11^	6.7267 × 10^–06^	0.9891	0.0837	3.7076 × 10^–11^	6.0890 × 10^–06^	0.9915
6–12–1	0.9875	0.0771	4.4745 × 10^–11^	6.6892 × 10^–06^	0.9875	0.0903	5.8320 × 10^–11^	7.6368 × 10^–06^	0.9858	0.0790	4.1049 × 10^–11^	6.4070 × 10^–06^	0.9907

Significant values are given in bold.

**Figure 4:**
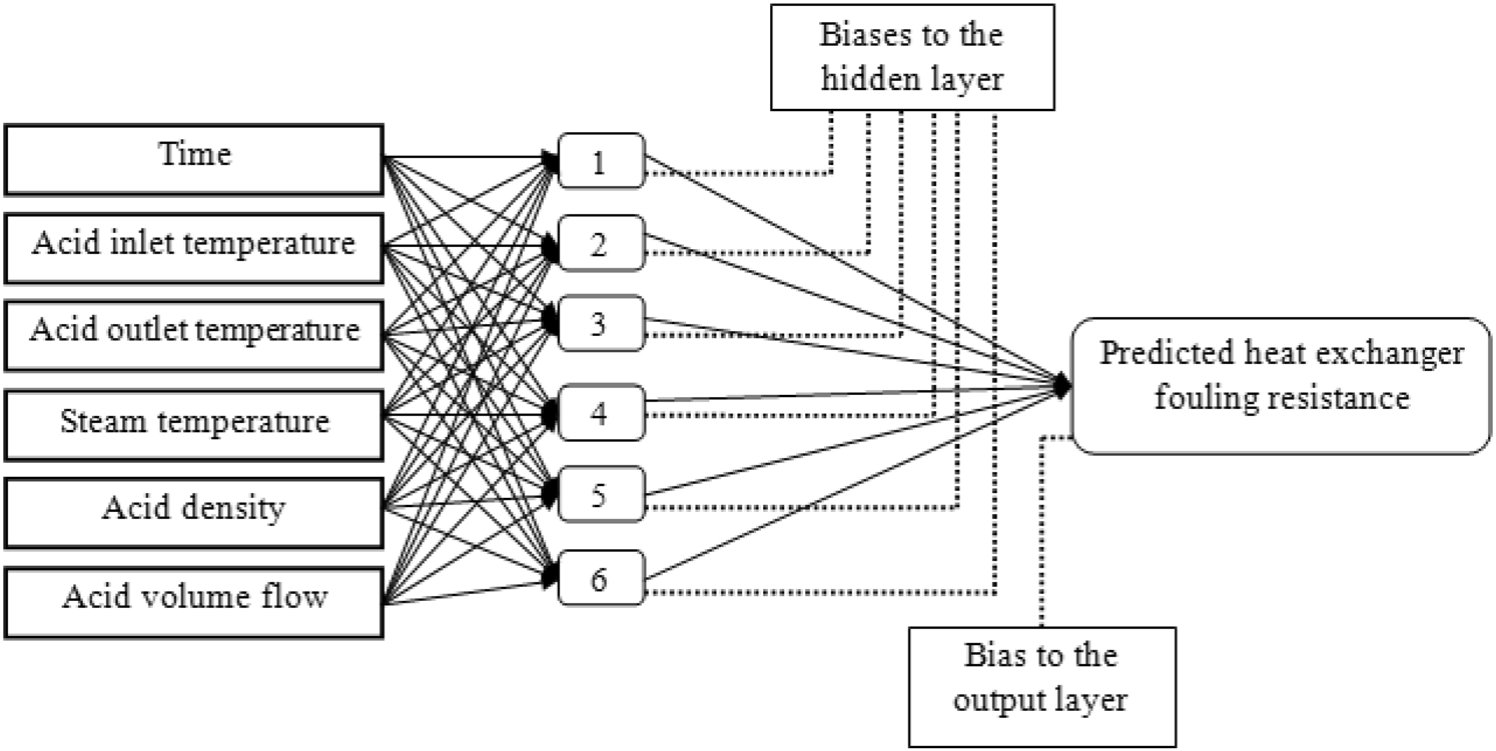
The optimal ANN configuration.

As can be seen from Table 6, when the number of hidden neurons increase to 6 in the training and validation processes, the values of AARD, MSE and RMSE of validation data set become smaller than other configurations while the value of r^2^ become higher. The adding of more neurons in the hidden layer may not improve the predicted results this is confirmed in the same table where the values of AARD, MSE and RMSE increase and the value of r^2^ decreases continuously. Therefore, for the studied heat exchanger, the optimal ANN configuration consists of six neurons in single hidden layer. Thus, in this case, ANN with configuration 6–6–1 is selected for estimation of the fouling resistance. The optimized network configuration is shown in Fig. 4.

For the validation data set, as can be seen from Table 6, the selected networks supplies a total of AARD = 0.0793%, MSE = 2.5853 10^–11^, RMSE = 5.0846 10^–06^ and r^2^ = 0.9938.

#### Appropriate ANN activation function and training algorithm

As mentioned in the previous section, based on the activation function, a comparison among the hyperbolic tangent transfer function, linear transfer function and sigmoid transfer function is carried out. Moreover, a comparison between the BP neural network with BFGS, Scaled Conjugate Gradient and Gradient Descent training functions is carried out.

The best transfer function and training algorithm are found by examining and comparing simultaneously these different algorithms. The selection is based on the values of the accuracy measurements. Consequently, various transfer functions and learning algorithms are training for each network having an optimal configuration. Table 7 presents the values of AARD %, MSE, RMSE and r^2^ in the training, validation and test datasets for the studied heat exchanger for three different transfer functions and algorithms. The choice of the best transfer function and training algorithm is based on the values of validation errors.

**Table 7:** Comparison of errors of various training algorithms for estimation of fouling resistance.

Transfer function	Algorithm	r^2^_ALL_	Training error				Validation error				Test error			
			AARD	MSE	RMSE	r^2^	AARD	MSE	RMSE	r^2^	AARD	MSE	RMSE	r^2^
Hyperbolic tangent	BFGS	**0.9950**	**0.0390**	**1.6685 × 10**^**–11**^	**4.0848 × 10**^**–06**^	**0.9952**	**0.0793**	**2.5853 × 10**^**–11**^	**5.0846 × 10**^**–06**^	**0.9938**	**0.0591**	**1.7071 × 10**^**–11**^	**4.1318 × 10**^**–06**^	**0.9958**
	Gradient descent	0.9833	0.0861	6.8332 × 10^–11^	8.2663 × 10^–06^	0.9820	0.0790	5.9663 × 10^–11^	7.7242 × 10^–06^	0.9865	0.0751	5.2096 × 10^–11^	7.2178 × 10^–06^	0.9865
	Conjugate gradient	0.9881	0.0601	4.0520 × 10^–11^	6.3655 × 10^–06^	0.9885	0.0956	5.6282 × 10^–11^	7.5021 × 10^–06^	0.9861	0.0877	4.4227 × 10^–11^	6.6503 × 10^–06^	0.9906
Linear	BFGS	0.8724	0.2892	4.2216 × 10^–10^	2.1028 × 10^–05^	0.8648	0.1315	3.3747 × 10^–10^	1.8370 × 10^–05^	0.9110	0.0967	4.5957 × 10^–10^	2.1437 × 10^–05^	0.8651
	Gradient descent	0.8034	0.3155	1.0365 × 10^–09^	3.2196 × 10^–05^	0.7861	0.2425	7.1870 × 10^–10^	2.6808 × 10^–05^	0.9118	0.3034	1.1865 × 10^–09^	3.4446 × 10^–05^	0.7351
	Conjugate gradient	0.8714	0.1087	4.4787 × 10^–10^	2.1163 × 10^–05^	0.8592	0.0936	3.2711 × 10^–10^	1.8086 × 10^–05^	0.9147	0.0936	4.6811 × 10^–10^	2.1635 × 10^–05^	0.8629
Sigmoid	BFGS	0.8707	0.0916	4.2463 × 10^–10^	2.0606 × 10^–05^	0.8668	0.0999	4.6469 × 10^–10^	2.1556 × 10^–05^	0.8747	0.0862	4.5130 × 10^–10^	2.1244 × 10^–05^	0.8669
	Gradient descent	0.8685	0.1068	4.4001 × 10^–10^	2.0976 × 10^–05^	0.8638	0.1170	4.5632 × 10^–10^	2.1361 × 10^–05^	0.8797	0.1062	4.7980 × 10^–10^	2.1904 × 10^–05^	0.8621
	Conjugate gradient	0.8684	0.0981	4.3499 × 10^–10^	2.0856 × 10^–05^	0.8640	0.1059	4.6255 × 10^–10^	2.1507 × 10^–05^	0.8760	0.0828	4.5455 × 10^–10^	2.1320 × 10^–05^	0.8654

Significant values are given in bold.

According to the accurate calculation, it is clear that the BFGS back-propagation with hyperbolic transfer function offers the best performance (the bold lines). For validation data, this algorithm presents the smallest values of AARD %, MSE and RMSE and the highest value of r^2^. Consequently, the BFGS back-propagation learning algorithm and the hyperbolic transfer function are simultaneously the most appropriate learning algorithm and activation function for the considered task. It provides the value r^2^_ALL_ = 0.9950 for the cross-flow heat exchanger.

#### Expression of the output parameter using ANN model

After selecting the optimal topology and finding the most appropriate activation function and training algorithm (Tables 6 and 7), the weight and biases values to construct our particular ANN model with configuration 6–6–1 are listed below:

$$w_{2,1} = \left[ {\begin{array}{*{20}c} {\begin{array}{*{20}r} \hfill {1.60223} \\ \end{array} } & {0.17246} & { - 0.39399} & {0.02347} & {0.35963} & { - 0.05585} \\ { - 1.38055} & { - 0.34202} & { - 0.18133} & { - 0.73088} & { - 0.44725} & {0.02337} \\ { - 1.13902} & {\begin{array}{*{20}r} \hfill { - 1.36280} \\ \end{array} } & {1.87377} & { - 0.42610} & {0.11855} & {0.25272} \\ { - 0.35060} & {0.68046} & { - 0.64473} & {0.08211} & {\begin{array}{*{20}r} \hfill {0.08260} \\ \end{array} } & { - 0.47503} \\ { - 2.10447} & {0.46459} & {0.56041} & {\begin{array}{*{20}r} \hfill { - 0.52793} \\ \end{array} } & { - 0.11390} & { - 0.58764} \\ {1.25291} & {0.20000} & { - 0.37410} & {0.76153} & {0.26310} & {0.23444} \\ \end{array} } \right]\;\;\;\;\;b_{2} = \left[ {\begin{array}{*{20}c} { - 0.50149} \\ { - 0.35202} \\ {0.91936} \\ {0.55180} \\ { - 0.75872} \\ {0.02690} \\ \end{array} } \right]$$

$$w_{3,2} = \left[ {\begin{array}{*{20}c} { - 0.39722} & { - 0.39638} & { - 2.19827} & {2.51059} & { - 0.95661} & { - 0.60076} \\ \end{array} } \right]\;\;\;b_{3} = \left[ {0.40541} \right]$$

Thus, our built model serves to predict the value of the fouling resistance of the cross-flow heat exchanger in the phosphoric acid concentration process. Therefore, the developed model is made up of a single hidden layer containing six neurons having hyperbolic tangent transfer function.

The predicted value of fouling resistance (R_f_) in real coordinates is calculated according to the following procedure:

1. All the independent variables should be normalized into an interval of [− 1 1] using Eq. (13) below^20^:
  13 $$\overline{d} = \left( {\frac{{d - d_{\min } }}{{d_{\max } - d_{\min } }}} \right) \times (n_{\max } - n_{\min } ) + n_{\min }$$
  where $\overline{d}$ refers to the normalized data values, d represents the numeric data value of each independent variable, d_min_ and d_max_ are the minimum and maximum data values, respectively, of each variable. n_min_ and n_max_ are the minimum and maximum values, respectively, of the new range. In our case n_min_ = − 1 and n_max_ =  + 1. Eq. (13) becomes^20^:
  14 $$\overline{d} = 2 \times \left( {\frac{{d - d_{\min } }}{{d_{\max } - d_{\min } }}} \right) - 1$$
  The matrix should be arranged in 6 rows and 1 column for the cross-flow heat exchanger.
2. Multiply the obtained normalized variables in the previous stage by the matrix of weights (w_2,1_) constituted by 6 rows and 6 columns.
3. Add the bias (b_2_) of the independent variables to the obtained results in stage 2.
4. Using hyperbolic tangent transfer function [Eqs. (15) and (16)] for calculating (y_j_) for the all six elements on the matrix of step 3^20^.
  15 $$y_{j} = f(t_{j} ) = \frac{{(e^{{(t_{j} )}} - e^{{( - t_{j} )}} )}}{{(e^{{(t_{j} )}} + e^{{( - t_{j} )}} )}}$$
  16 $$t_{j} = \sum\limits_{r = 1}^{k} {(w_{j,r} } x_{r} + b_{j} )$$
  where t_j_ is the threshold. The input which is multiplied by the corresponding weights (w_j,r_x_r_), summed together, added extra bias (b_j_) and applied to an activation or transfer function (f) to form a single output (y_j_).
5. Multiply the results obtained in step 4 (Matrix size = (6 × 1)) by the transpose of the weight of the dependent variable (w_3, 2_).
6. Add the value of the bias (b_3_) to the result of step 5 (i.e. 0.40541).
7. Calculate (y_j_) for the limited predicted value of fouling resistance (R_f_) obtained in the previous step using (Eq. 15).
8. The predicted value of the fouling resistance (R_f_) obtained is calculated according to the following relationships:
  17 $$R_{f}^{pred} = y_{j} \times \left( {\frac{{R_{f\;\max } - R_{f\;\min } }}{2}} \right) - R_{f\;\min }$$

Noted that the parameters of our intelligent model are adjusted by experimental data of the considered system. The construction of an intelligent system with good estimation of fouling resistance is for a specific type of fluid which is the phosphoric acid. It is applicable to a whole of variables within the allowable ranges as shown in Table 5. For operating periods ranging up to 122 h, the inlet and outlet temperatures of fluid and the steam temperature should not exceed 78 °C, 86.8 °C and 125 °C, respectively, the phosphoric acid density should not be above 1656 kg/m^3^, and the volume flow rate should not be below 2102 m^3^/h to obtain values of (R_f_) close to reality by the developed model.

#### ANN model evaluation

**Figure 5:**
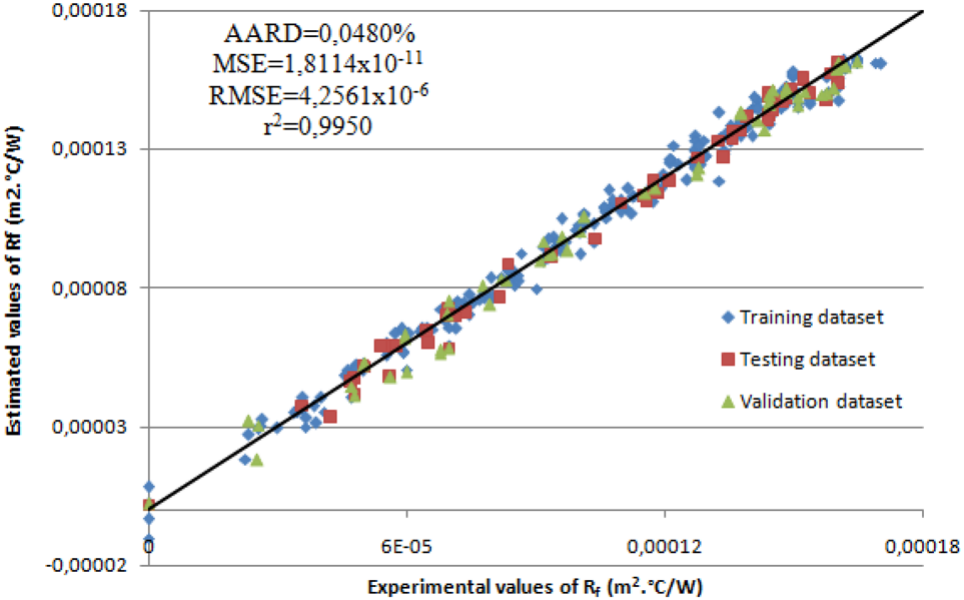
ANN estimation versus experimental results.

To check the adequacy of the ANN model, a comparison between the experimental data and the estimated data is depicted in Fig. 5. In this case, estimated results were found close to the experimental results. The concentration of the experimental points around the 45° line affirms that the data are well-fitted.

Table 6 display the simulation values obtained from the optimized ANN model. The value of the correlation coefficient (r^2^_ALL_) of the simulated data versus the experimental data of fouling resistance was 0.9950 (r^2^_validation_ = 0.9938, r^2^_training_ = 0.9952 and r^2^_test_ = 0.9958). If (r^2^) values are higher than 0.9, this indicate a good relationship amid the experimental and predicted values^20^. In this case, the constructed ANN model, which was trained using experimental values, estimated the fouling resistance efficiently in cross-flow heat exchanger. For this reason, the use of the ANN to estimate the performance of thermal systems in engineering applications is recommended.

### Comparison of the estimated values using RSM and ANN models

**Figure 6:**
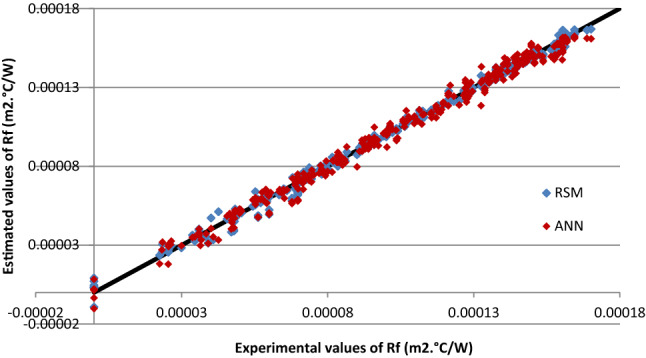
Comparison of experimental values with RSM and ANN estimation of fouling resistance.

In this research, the thermal performance of cross-flow heat exchanger was estimated by the RSM and ANN methods, as detailed in the previous sections (“Response surface methodology” and “Artificial Neural Network”). Furthermore, the expressions of the fouling resistance were derived. To evaluate the accuracies of the developed ANN and RSM models, the estimated parameter was compared with the experimental data, which is displayed in Fig. 6.

From this regression diagram of the ANN and RSM estimated models, data of RSM model was found nearly distributed on 45° line than ANN model.

In addition, five statistical parameters were selected for comparison to further verify the estimation models, as shown in Table 8. This statistical investigation showed that the r^2^ estimated by RSM is more accuracy, and the value is comparatively closer to 1 than ANN approach. This involves that model developed by RSM was more effective and estimated the fouling resistance more precisely. The lowest values of RMSE, MSE and RMSE for RSM than of ANN confirm that the RSM has also clear perfection comparing to ANN.

**Table 8:** Comparison between RSM and ANN.

Parameter	RSM	ANN
AARD	0.0397	0.0480
MSE	8.2525 × 10^–12^	1.8114 × 10^–11^
RMSE	2.8727 × 10^–6^	4.2561 × 10^–6^
r^2^	0.9976	0.9950

According to the above-discussed viewpoints, RSM yields a more reasonable performance than ANN in estimating the fouling resistance.

A similar scenario with the result found in this study has also been observed by Awolusi et al.^50^ which proved that the RSM model is more accurate than the ANN model. In contrast, several authors discovered a better precision of ANN model versus to RSM model.

For this reason, the result offers the prospect of RSM may also precede in estimation over ANN with better accuracy. This also ceases the former beliefs of ANN being invariably better.

## Conclusions

In this paper, two efficient and highly accurate estimation tools which are response surface methodology and artificial neural network were applied to analyze and estimate the thermal performance of cross-flow heat exchanger with the aim of evaluating their suitability for application in the cleaning schedule of the heat exchanger and for controlling operation of the phosphoric acid concentration plant. The influences and significances of the process parameters on the fouling resistance were analyzed by adopting the analysis of variance as the statistical procedure. By using these two models, the relationship between the process parameters and response was determined.

The main results derived from this work are the following:

- The regression model of fouling resistance was developed and evaluated for accuracy during the analysis of RSM model. Six sets of interactions of the operating variables of the concentration loop that had significant effects on the fouling resistance were studied in detail. The value of r^2^ was closely to 1 (r^2^ = 0.9976). The AARD of the fouling resistance was 0.0397% and the MSE and the RMSE were less than 0.00028%. The values of statistical parameters indicate that the model had good accuracy and the regression diagram show that good fit was achieved. The selected input parameters had statistically significant impacts on the thermal performance of heat exchanger increased with the acid inlet temperature and decreased with acid outlet temperature and volume flow rate.
- An ANN model based on back-propagation algorithm was developed after using 70% of the experimental data for training. After the process of training, validation and testing, it was found that model of BFGS 6–6–1 was the best architecture for fouling resistance. The value of r^2^ was closely to 1 (r^2^_ALL_ = 0.9950). The AARD of the fouling resistance was 0.0591% during testing process and the MSE and the RMSE were less than 0.0004% for the training process indicating that the predictive results of the network were in good agreement.
- The statistical parameters were also used to compare the experimental and estimated values for both methods while the visual inspection of the regression diagram was used to further verify and compare the estimation models. The results of the statistical parameters indicated that the r^2^ value estimated by RSM is more accuracy, and the value is comparatively closer to 1 than ANN approach. The values of AARD, MSE and RMSE for RSM are less than of ANN. In addition, the visual inspection showed that the estimated values obtaining by RSM and experimental values were nearly distributed on 45° line. All these results confirm that the RSM model is more accurate than the ANN model.
